# Knee Septic Arthritis Caused by Coinfection with *Rothia mucilaginosa* and *Erysipelothrix rhusiopathiae*

**DOI:** 10.3390/antibiotics14090880

**Published:** 2025-08-31

**Authors:** Danguole Vaznaisiene, Matas Simkus, Edita Druzaite, Justinas Stucinskas, Pranciskus Bakutis

**Affiliations:** 1 Department of Infectious Diseases, Medical Academy, Lithuanian University of Health Sciences, LT-47116 Kaunas, Lithuania; 2 Faculty of Medicine, Medical Academy, Lithuanian University of Health Sciences, LT-44307 Kaunas, Lithuania; matas.simkus@stud.lsmu.lt (M.S.);; 3 Department of Orthopaedics and Traumatology, Medical Academy, Lithuanian University of Health Sciences, LT-50161 Kaunas, Lithuania; justinas.stucinskas@lsmu.lt (J.S.); pranciskus.bakutis@lsmu.lt (P.B.)

**Keywords:** septic arthritis, *Erysipelothrix rhusiopathiae*, *Rothia mucilaginosa*

## Abstract

**Introduction**: Septic arthritis of the knee caused by the combination of *Rothia mucilaginosa* and *Erysipelothrix rhusiopathiae* is extremely rare. *E. rhusiopathiae* is a rare zoonotic pathogen that primarily affects individuals with occupational exposure to animals, while *R. mucilaginosa* can cause severe infections, particularly in immunocompromised patients. **Case Presentation**: A 59-year-old male underwent right knee arthroscopy in 2019 due to meniscal degeneration. Two weeks later, activity-related pain appeared. Magnetic resonance imaging showed proliferative synovitis, and joint aspiration revealed the presence of *E. rhusiopathiae*, which was treated with ciprofloxacin. As inflammation persisted, arthroscopic synovectomy was performed. Cultures revealed *R. mucilaginosa* and *E. rhusiopathiae*, prompting treatment with vancomycin and clindamycin. Despite repeated synovectomies, symptoms remained. After knee trauma in 2023, infection recurred. A two-stage total knee arthroplasty was performed in early 2024. At this time, another pathogen was isolated. At 12-month follow-up, the patient’s function and alignment had improved significantly. **Conclusions**: The described case highlights the importance of anamnesis, early diagnostics, and knowledge about the possible resistances of rare pathogens to ensure appropriate treatment of the illness.

## 1. Introduction

Septic arthritis of the knee is a serious infectious condition that requires prompt diagnosis and intervention to prevent irreversible joint damage and functional impairment. While *Staphylococcus aureus* remains the most common causative organism, specific pathogens such as *Erysipelothrix rhusiopathiae* or *Rothia mucilaginosa* are rarely implicated [1,2,3,4]. *R. mucilaginosa* typically affects immunocompromised individuals, while *E. rhusiopathiae* affects those with specific occupational exposures. Infections caused by these pathogens may follow an indolent or atypical clinical course, often with low-grade systemic inflammatory responses, complicating early diagnosis. 

The most common form of human infection caused by *E. rhusiopathiae* is the localized cutaneous form, known as erysipeloid [5]. Systemic infection with bacteremia is relatively uncommon and is often complicated by endocarditis. Very rare forms of *E. rhusiopathiae* infection include neuroinfections, ocular infections, intra-abdominal infections, soft tissue infections, bone and joint infections, periprosthetic joint infections, and pulmonary infections [5,6,7]. 

Bacteremia is the most common infection caused by *R. mucilaginosa*, particularly in immunocompromised patients. However, it can also cause other very rare infections similar to those caused by *E. rhusiopathiae*, including neurologic, skin and soft tissue, bone and joint, periprosthetic joint, and pulmonary and intra-abdominal infections, endocarditis [8].

The literature contains only a limited number of case reports describing septic arthritis caused by these organisms, particularly involving the native knee joint in immunocompetent adults. Accurate identification and effective treatment are further challenged by their uncommon antimicrobial susceptibility profiles and the potential need for surgical intervention [9]. In this article, we present a case of septic arthritis caused by coinfection with *E. rhusiopathiae* and *R. mucilaginosa* in a patient without immunosuppression or contact with animals. To the best of our knowledge, no similar case has been reported in the literature.

## 2. Case Presentation

**Patient history and initial findings:** On 24 May 2019, a 59-year-old male patient underwent right knee arthroscopy due to degenerative changes of the meniscus. After two weeks, the patient started to feel pain during physical activities. 

**Diagnosis:** Blood tests revealed leucocytosis (Table 1), while MRI (magnetic resonance imaging) showed proliferative synovitis. On 2 March 2020, knee arthrocentesis showed right knee joint inflammation (Table 2), and culture revealed *E. rhusiopathiae* susceptible to penicillin and ciprofloxacin (Table 3). Ciprofloxacin was started for 2 weeks, but signs of inflammation persisted. On 16 May 2020, the patient underwent arthroscopic synovectomy due to persistent synovitis.

**Empirical antimicrobial treatment:** Intraoperatively, ampicillin–sulbactam was administered at a dose of 1.5 g, four times daily empirically, which was continued in the postoperative period. However, the patient developed a probable allergic reaction to ampicillin–sulbactam, requiring a switch to vancomycin (1 g, twice daily). 

**Targeted antimicrobial treatment**: Intraoperative cultures revealed two rare pathogens—*R. mucilaginosa* and *E. rhusiopathiae*—identified via matrix-assisted laser desorption/ionization–time-of-flight (MALDI-TOF) mass spectrometry. The antimicrobial susceptibility results were obtained in a certified laboratory. EUCAST clinical breakpoints for non-species-related bacteria were used [10] (Table 4). The method used was Etest, in accordance with the laboratory’s standard operating procedures. Following these findings, clindamycin (300 mg, three times daily) was added intravenously to vancomycin for 4 weeks, after which the patient was switched to oral clindamycin for 4 weeks. This treatment was intended to ensure good bone penetration and provide bi-therapy against Gram-positive cocci in the management of bone and joint infection.

**Figure 1:**
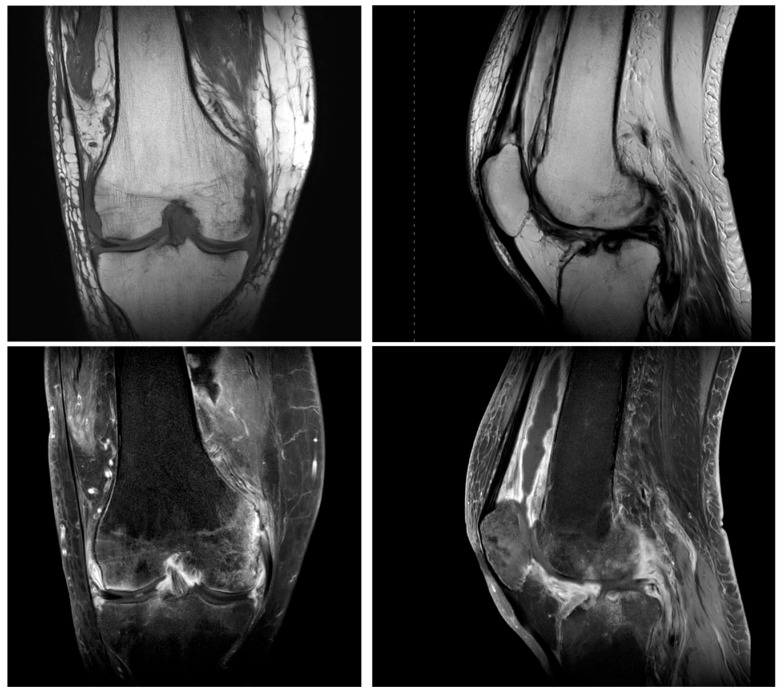
Magnetic resonance imaging showed inflammatory changes in the setting of underlying osteoarthritis.

**Figure 2:**
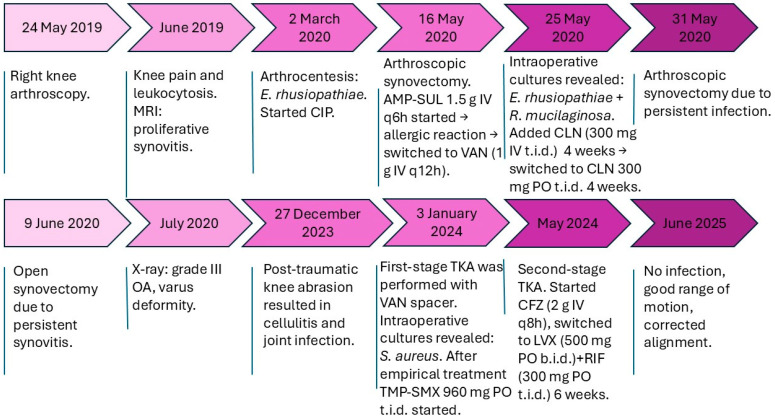
Timeline of clinical events. Abbreviations: AMP-SUL, ampicillin–sulbactam; CIP, ciprofloxacin; CLN, clindamycin; VAN, vancomycin; TMP-SMX, trimethoprim–sulfamethoxazole; CFZ, cefazolin; LVX, levofloxacin; RIF, rifampicin; OA, osteoarthritis; MRI, magnetic resonance imaging; TKA, total knee arthroplasty.

**Decision on arthroplasty:** Despite the initial arthroscopic synovectomy performed on 16 May, a repeat arthroscopic procedure was required on 31 May due to persistent joint inflammation, which was followed by an open synovectomy on 9 June as clinical symptoms of infection and synovitis remained refractory to prior surgical and antibacterial treatments. After all these surgical procedures, MRI showed fluid accumulation, inflammatory changes and avascular necrosis of the lateral condyle of the femur (Figure 1). The knee had flexion and extension deficit and varus deformity. The X-ray showed grade III osteoarthrosis with varus deformity. On 27 December 2023, the patient sustained trauma to the knee due to a fall from standing height, resulting in soft tissue injury and subsequent joint involvement, likely leading to cellulitis. Synovial fluid analysis indicated infection. The decision was made to perform a two-stage TKA (total knee arthroplasty) due to suspected infection and underlying osteoarthritis. On 3 January 2024, first-stage TKA was performed, and an MOLD (temporary cemented joint spacer) with vancomycin was implanted. Culture revealed *Staphylococcus aureus* and, after empirical treatment with ampicillin–sulbactam and vancomycin, trimethoprim–sulfamethoxazole was prescribed. At 4 months later, there were no more signs of infection and second-stage TKA was completed following 6 weeks of antibacterial therapy: initial treatment with cefazolin, followed by levofloxacin in combination with rifampicin. The surgery was successful, flexion and extension were increased, and the leg axis was normal, yielding good results after 12 months of follow-up (Table 5, Figure 2).

## 3. Discussion

Septic arthritis caused by *E. rhusiopathiae* or *R. mucilaginosa* is rare and may present with less-pronounced clinical manifestations and milder elevations of inflammatory markers, which can potentially delay diagnosis. In contrast, *S. aureus*—a well-recognized predominant pathogen in septic arthritis—typically causes a far more acute presentation with pronounced clinical and laboratory signs of inflammation, including markedly elevated C-reactive protein (CRP) and white blood cell (WBC) counts, evident joint inflammation, and characteristic changes in synovial fluid color and viscosity, often accompanied by severe symptoms. 

The incidence of septic arthritis is estimated at two to six cases per 100,000 individuals annually, with increased rates observed in the presence of risk factors such as advanced age, diabetes mellitus, rheumatoid arthritis, joint prostheses, recent arthroplasty or intra-articular injections, cutaneous infections or ulcers, and osteoarthritis [11]. Septic arthritis of the knee is a potentially life-threatening condition that necessitates urgent medical intervention. Management includes the prompt initiation of empirical intravenous antimicrobial therapy following joint aspiration and culture collection, alongside appropriate joint drainage—via arthrotomy, arthroscopy or repeated needle aspiration—to control the infection and preserve joint function [12].

It has been reported that *E. rhusiopathiae* septic arthritis typically presents as a chronic process involving a single big joint, particularly the knee. *E. rhusiopathiae* is most cited in the literature as a causative agent of prosthetic knee joint infections; however, in the literature, we found a clinical case in which native knee septic arthritis developed following an arthroscopic knee joint surgery. Furthermore, only a few reports have described moderate fever [13]. This pathogen should be suspected if there is a history of animal contact, especially if the patient works on farms, raises swine or is a fisherman. Interestingly, our patient had no known contact with animals and was not a fisherman.

Despite their relatively low inherent virulence, *Rothia* species have been recognized as opportunistic pathogens that are capable of causing severe infections, particularly in immuno-compromised patients such as those with hematologic malignancies and neutropenia [1,11]. In this case, the patient did not have any underlying conditions associated with immunosuppression that would facilitate the suspicion of septic arthritis of the knee joint caused by this bacterium. Furthermore, *Rothia* species are frequently associated with poor dental hygiene, periodontal disease and mucosa-compromising conditions which may predispose individuals to bacteremia and subsequent joint or prosthetic infections. Given this uncertainty, oral health assessment should be considered an important part of the preoperative evaluation, especially prior to joint operations. For patients with evidence of poor oral hygiene or mucosal disease, dental consultation and targeted antibiotic prophylaxis may be a prudent preventive strategy [9,14,15]. Our patient had no history of dental procedures or periodontal disease within the past year. 

Although efforts were made to determine the source of these pathogens in our case, the exact origin remained unidentified. The patient did, however, present several known risk factors for joint infection, including prior knee arthroscopy, repeated intra-articular aspirations and underlying knee osteoarthritis.

In this case report, both *E. rhusiopathiae* and *R. mucilaginosa* were identified in the affected knee joint. Although both organisms may act synergistically in the pathogenesis of the disease, the clinical data from a single case do not permit distinguishing the specific contribution of each species. Further insight could be gained through comparative analyses of cases with single-species versus mixed infections. While evaluating individual case reports according to the need for repeated interventions, it appears that *R. mucilaginosa* may have played a more substantial role in the disease’s severity than *E. rhusiopathiae* (Table 6). In the literature, three cases of *E. rhusiopathiae* and one case of *R. mucilaginosa* septic monoarthritis of the native knee joint have been reported in immunocompetent adults (i.e., without moderate or severe immunosuppression) (Table 6). To the best of our knowledge, no cases describing coinfection with both of these rare pathogens have been documented in the literature.

The primary diagnostic method for septic arthritis is the analysis of synovial fluid obtained from the affected joint, which includes culture, Gram stain, crystal analysis and WBC count with differential. A synovial fluid WBC count exceeding 50,000 cells/μL with a predominance of neutrophils (>90%) strongly suggests a bacterial etiology [16]. The detection of a bacterial pathogen in synovial fluid conclusively establishes the diagnosis of septic arthritis. Additional diagnostic tests include a complete blood count (CBC), erythrocyte sedimentation rate (ESR), CRP levels and blood cultures. The peripheral blood WBC count is often elevated, typically accompanied by a left shift. While increased ESR and CRP levels support the diagnosis, they are not definitive [12]. In both infections, inflammatory markers were frequently low, which rendered the identification of causative pathogens difficult as the septic arthritis of the knee joint followed an atypical clinical course, with only a few cases caused by these specific bacteria having been reported in the literature [1]. In our case, as well as in the cases reported in the literature, a possible exacerbation of osteoarthritis played an important role in the differential diagnosis of infection.

The management of septic arthritis involves the prompt initiation of antimicrobial therapy in conjunction with adequate joint drainage, which may be achieved through arthrotomy, arthroscopy or serial needle aspiration. Empirical intravenous antibacterial therapy should commence immediately after synovial fluid aspiration and culture collection. Initial empirical regimens should provide antistaphylococcal coverage [12,17]. In our reported case, the standard empirical treatment used in our institution for orthopedic infections was prescribed to the patient.

Both of the considered pathogens can be resistant to some commonly used antibiotics, which can make it difficult to treat these rare infections [11]. *E. rhusiopathiae* infections are typically treated with penicillin, although alternative antibiotics such as cephalosporins, clindamycin or fluoroquinolones can also be used; meanwhile, *R. mucilaginosa* infections can be treated with beta-lactams or vancomycin, including combined therapy [5,8]. Initial cultures isolated *E. rhusiopathiae*, which demonstrated susceptibility to penicillin and ciprofloxacin. Based on these findings, ciprofloxacin therapy was initiated. Although cases successfully treated with penicillin have been reported in the literature, ciprofloxacin was chosen due to its good bone penetration. Despite treatment, the patient’s inflammatory symptoms persisted. A subsequent arthroscopic synovectomy was performed, during which both *E. rhusiopathiae* and *R. mucilaginosa* were isolated. The identification of a new pathogen could explain the lack of response to ciprofloxacin, to which the latter pathogen was resistant. Although *E. rhusiopathiae* initially demonstrated susceptibility to both penicillin and ciprofloxacin, the same organism isolated following ciprofloxacin monotherapy exhibited penicillin resistance while retaining susceptibility to ciprofloxacin. This change may be explained by co-selection of resistance. Vancomycin and clindamycin were added to broaden antimicrobial coverage, ensuring good bone penetration and providing bi-therapy against Gram-positive cocci for management of the bone and joint infection; however, clinical signs of infection continued. *S. aureus* was identified as a pathogen more than three years after the initial infection with *R. mucilaginosa* and *E. rhusiopathiae*. This likely represents a reinfection, rather than a continuation of the initial infection. Additionally, the patient had concomitant osteoarthritis, which could have contributed to the symptoms observed.

Due to signs of infection, necrosis and advanced osteoarthritis, the patient ultimately underwent two-stage total knee arthroplasty. This procedure had not been previously performed in this patient due to efforts to preserve the native knee joint and other commonly reported reasons, such as the patient’s relatively good joint function at the time, the absence of advanced radiographic changes and the initial expectation that infection could be eradicated with debridement and targeted antibacterial therapy.

**Table 6:** Cases of *Rothia mucilaginosa* and *Erysipelothrix rhusiopathiae* septic monoarthritis of the native knee joint in immunocompetent (i.e., without severe or moderate immunosuppression) adults reported in the literature.

	***Erysipelothrix rhusiopathiae***	***Rothia*** ***mucilaginosa***	**Present Case (Coinfection)**
**Number of reported cases (authors)**	3 (Ruiz ME 2003 [2]; Vallianatos PG 2003 [13]; Neumann DRP 2009 [18])	1 (Daoub A 2021 [4])	1 (present case)
**Demographics**	Male, 18–76 years old	Female, 58 years old	Male, 59 years old
**Epidemiological history**	(1) Gardening and fishing; (2) 8 days after an arthroscopic anterior cruciate ligament reconstruction; (3) Farmer, osteoarthritis.	Bilateral knee osteoarthritis treated with osteotomy and intra-articular steroid injections (at 12 months and 3 days before admission), dental procedure (2 weeks before symptoms)	Knee arthroscopy due to pathology of the meniscus, osteoarthritis
**Microorganisms**	*E. rhusiopathiae*	*R. mucilaginosa*	*E. rhusiopathiae + R. mucilaginosa*
**Antibacterial treatment and duration**	(1) Penicillin IV 4 weeks; (2) IV 6 weeks -> oral 16 weeks; (3) Penicillin IV 3 weeks -> amoxicillin–clavulanate 3 weeks.	Empirical vancomycin -> flucloxacillin and co-trimoxazole IV 2 weeks -> oral linezolid	Empirical vancomycin -> vancomycin and clindamycin IV 4 weeks -> oral clindamycin 4 weeks (6 weeks after last surgery)
**Surgical interventions (number)**	(1) Repeated knee arthrocentesis; (2) Arthroscopic lavage and debridement; (3) Arthroscopic debridement -> total knee arthroplasty 6 months after the first presentation and recovery (for concomitant osteoarthritis).	Arthrocentesis, three arthroscopic lavages, surgical treatment to treat osteoarthritis will be considered at 1 year post-discharge	Two arthroscopic synovectomies, one open synovectomy -> two-stage total knee arthroplasty for infection (*S. aureus*) and concomitant osteoarthritis
**Outcome**	Full recovery in all cases	Full recovery	Full recovery

The presented clinical case has a limitation: the culture was not preserved for imaging of the test results, although the culture is clearly depicted in the literature sources.

## 4. Conclusions

The described case highlights the importance of anamnesis, early diagnostics, and knowledge about the possible resistances of rare pathogens to ensure appropriate treatment of the illness. This report demonstrates the complexity of managing chronic joint infections, especially when rare pathogens such as *E. rhusiopathiae* and *R. mucilaginosa* are involved. Despite multiple surgical procedures and changes in antibiotic therapy due to allergic reactions and persistent infection, successful TKA was eventually achieved. 

## Figures and Tables

**Table 1:** Dynamics of blood tests.

Test/Date	27 June 2019	20 January 2020	2 March 2020	15 May 2020
CRP (mg/L)	15.6	7.3	32.8	60.5
WBC (×10^9^/L)	10.9	12.8	8.7	13.2
NE (%)	82.0	86.6	61.5	82.3
NE (×10^9^/L)	9.0	11.1	5.4	10.9
ESR (mm/h)	62	28	—	—
PLT (×10^9^/L)	439	484	598	670

Abbreviations: WBC, white blood cells; CRP, C-reactive protein; NE, neutrophils; ESR, erythrocyte sedimentation rate; PLT, platelets.

**Table 2:** Dynamics of synovial fluid analysis.

Test/Date	20 January 2020	2 March 2020	15 May 2020
WBC (×10^9^ L)	17.76	41.6	13.7
RBC (×10^12^/L)	0.001	0.056	0.003
MN (%)	55.0	62.0	10.0
MN (×10^6^/L)	9.77	25.7	13.7
PMN (%)	45.0	38.0	90.0

Abbreviations: WBC, white blood cells; RBC, red blood cells; MN, mononuclear cells; PMN, polymorphonuclears.

**Table 3:** *Erysipelothrix rhusiopathiae* susceptibility profile (synovial fluid).

Antibiotic/Microorganism	*Erysipelothrix rhusiopathiae*
Penicillin	S
Cefuroxime	S
Ciprofloxacin	S
Clindamycin	S
Gentamicin	R
Vancomycin	R

Abbreviations: S, susceptible; R, resistant.

**Table 4:** *Erysipelothrix rhusiopathiae* and *Rothia mucilaginosa* susceptibility profile (intraoperative cultures).

Antibiotic/Microorganism	*Erysipelothrix rhusiopathiae*	*Rothia mucilaginosa*
Penicillin	R	
Oxacillin		S
Cefuroxime	S	
Ciprofloxacin	S	R
Clindamycin	S	S
Gentamicin	R	R
Vancomycin	R	S
Trimethoprim/sulfamethoxazole		R
Rifampicin		S

Abbreviations: S, susceptible; R, resistant.

**Table 5:** Dynamics of blood parameters after second-stage TKA.

Test/Date	8 January 2024	19 January 2024	26 January 2024	30 January 2024	2 February 2024	6 February 2024	12 February 2024
CRP (mg/L)	128.8	55.4	33.8	78.1	42.8	10.4	10.4
RBC (×10^12^/L)	2.92	2.96	3.08	2.89	3.02	3.15	3.36
WBC (×10^9^/L)	13.6	11.4	7.6	5.9	6.7	7.8	7.6
NE (×10^9^/L)	10.5	7.5	4.2	3.9	3.4	3.2	3.2
NE (%)	77.2	65.5	55.2	66.0	50.6	41.6	41.5
PLT (×10^9^/L)	709	410	640	477	347	346	592

Abbreviations: WBC, white blood cells; CRP, C-reactive protein; NE, neutrophils; PLT, platelets; RBC, red blood cells.

## Data Availability

Dataset available on request from the authors.

## Abbreviations

The following abbreviations are used in this manuscript:

MRI	Magnetic resonance imaging
TKA	Total knee arthroplasty
WBC	White blood cells
RBC	Red blood cells
CRP	C-reactive protein
NE	Neutrophils
ESR	Erythrocyte sedimentation rate
PLT	Platelets
MN	Mononuclear cells
PMN	Polymorphonuclears
AMP-SUL	Ampicillin–sulbactam
CIP	Ciprofloxacin
CLN	Clindamycin
VAN	Vancomycin
TMP-SMX	Trimethoprim–sulfamethoxazole
CFZ	Cefazolin
LVX	Levofloxacin
RIF	Rifampicin
OA	Osteoarthritis

## References

[1] Janse T.S., Schermerhorn D.F., Colantonio D.T., Larson R.J., McGill R.J. (2023). A case of periprosthetic joint infection because of *Rothia mucilaginosa*. Mil. Med..

[2] Ruiz M.E., Richards J.S., Kerr G.S., Kan V.L. (2003). *Erysipelothrix rhusiopathiae* septic arthritis. Arthritis Rheum..

[3] Maillard A., Wakim Y., Itani O., Ousser F., Bleibtreu A., Caumes E., Monsel G. (2021). Osteoarticular infections caused by *Erysipelothrix rhusiopathiae*: Case report and literature review. Open Forum Infect. Dis..

[4] Daoub A., Ansari H., Orfanos G., Barnett A. (2021). *Rothia mucilaginosa*: A case of septic arthritis in a native knee and review of the literature. BMJ Case Rep..

[5] Veraldi S., Girgenti V., Dassoni F., Gianotti R. (2009). Erysipeloid: A review. Clin. Exp. Dermatol..

[6] Elvy J., Hanspal I., Simcock P. (2008). A case of *Erysipelothrix rhusiopathiae* causing bilateral endogenous endophthalmitis. J. Clin. Pathol..

[7] Meric M., Ozcan S.K. (2012). *Erysipelothrix rhusiopathiae* pneumonia in an immunocompetent patient. J. Med. Microbiol..

[8] Ekkelenkamp M.B., Rooijakkers S.H.M., Bonten M.J.M., Cohen J., Opal S.M., Powderly W.G. (2010). Chapter 165—Staphylococci and micrococci. Infectious Diseases.

[9] Verrall A.J., Robinson P.C., Tan C.E., Mackie W.G., Blackmore T.K. (2010). *Rothia aeria* as a cause of sepsis in a native joint. J. Clin. Microbiol..

[10] The European Committee on Antimicrobial Susceptibility Testing (EUCAST) Guidance: What to Do When There Are No Breakpoints; EUCAST: Växjö, Sweden, Revised February 2024. Guidance: What to Do When There Are No Breakpoints.

[11] Ramanan P., Barreto J.N., Osmon D.R., Tosh P.K. (2014). Rothia bacteremia: A 10-year experience at Mayo Clinic, Rochester, Minnesota. J. Clin. Microbiol..

[12] Momodu I.I., Savaliya V. (2023). Septic Arthritis. StatPearls [Internet].

[13] Vallianatos P.G., Tilentzoglou A.C., Koutsoukou A.D. (2003). Septic arthritis caused by *Erysipelothrix rhusiopathiae* infection after arthroscopically assisted anterior cruciate ligament reconstruction. Arthroscopy.

[14] Trivedi M.N., Malhotra P. (2015). Rothia prosthetic knee joint infection. J. Microbiol. Immunol. Infect..

[15] Mahobia N., Chaudhary P., Kamat Y. (2013). Rothia prosthetic knee joint infection: Report and mini-review. New Microbes New Infect..

[16] Margaretten M.E., Kohlwes J., Moore D., Bent S. (2007). Does this adult patient have septic arthritis?. JAMA.

[17] Clerc O., Prod’hom G., Greub G., Zanetti G., Senn L. (2011). Adult native septic arthritis: A review of 10 years of experience and lessons for empirical antibiotic therapy. J. Antimicrob. Chemother..

[18] Neumann D.R., Hafner M., Dorn U. (2009). Kniegelenksinfekt mit *Erysipelothrix rhusiopathiae*: Fallbericht und Übersicht der internationalen Fachliteratur. Z. Orthop. Unfall..

